# The complete chloroplast genome sequence of *Camellia chuongtsoensis*

**DOI:** 10.1080/23802359.2020.1856009

**Published:** 2021-01-27

**Authors:** Bo Yu, Ying-Bo Sun, Li-Li Huang, Ye-Chun Xu, Chao-Yi Zhao, Xiao-Fei Liu

**Affiliations:** Environmental Horticulture Institute, Guangdong Academy of Agricultural Sciences, Guangdong Key Lab of Ornamental Plant Germplasm Innovation and Utilization, Key Laboratory of Urban Agriculture in South China, Ministry of Agriculture, Guangzhou, Guangdong, China

**Keywords:** Chloroplast genome, Illumina sequencing, *Camellia chuongtsoensis*

## Abstract

*Camellia chuongtsoensis* is an evergreen shrub with a single-petaled flower and golden yellow color. The complete chloroplast genome of *C. chuongtsoensis* was sequenced and analyzed in this study by Illumina sequencing. The chloroplast genome is 156,504 bp in length with a quadripartite structure containing a large single copy (LSC) region of 86,215 bp, a small single copy (SSC) region of 18,253 bp, and a pair of inverted repeat regions of 26,018 bp (IRa and IRb). The chloroplast genome of *C. chuongtsoensis* encodes 135 genes, comprising 87 protein-coding genes, 37 tRNA genes, 8 rRNA genes, and 3 pseudogenes.

*Camellia chuongtsoensis*, a newly discovered species, set out bright yellow flowers (Li et al. 2018), which belongs to the genus *Camellia* in the family Theaceae. *C. chuongtsoensis* was planted in the Environmental Horticulture Research Institute of the Guangdong Academy of Agricultural Sciences (N23°23′, E113°23′, Guangzhou, China) (No: EHRIGAASC001). *Camellia chuongtsoensis* had a unique and long blooming period across the summer season (Li et al. 2018), therefore, it has great horticultural application value and is a good parent source in breeding of *Camellia*.

The chloroplast genome DNA of *C. chuongtsoensis* was extracted from young leaves. Using Covaris M220 (Covaris, Woburn, MA) for breaking the DNA into 300 bp fragments, we constructed shotgun sequencing libraries according to the TruSeq™ DNA Sample Prep Kit for Illumina. Finally, whole genome sequencing was executed using the Illumina NovaSeq platform (Illumina, USA) (Genepioneer Biotechnologies Co. Ltd, Nanjing, China). Pair-end Illumina raw reads were cleaned from adaptors and barcodes and then quality filtered using Trimmomatic (Bolger et al. 2014). Then, reads were mapped to the chloroplast genome of the reference species (Genbank accession number: NC_024663). Bowtie2 v2.2.4 (Langmead and Salzberg 2012) was used to exclude reads of nuclear and mitochondrial origins. Using SPAdes 3.6.1(Bankevich et al. 2012) to reconstruct the chloroplast genomes by *de novo* assembly, and chloroplast contigs were concatenated into larger contigs using Sequencher 5.3.2 (Gene Codes Inc., Ann Arbor, MI). A “genome walking” technique was used to remove gaps (Souza et al. 2019). Misassembled contigs were corrected by Jellyfish v.2.2.3 (Marcais and Kingsford 2011). Annotation of the chloroplast genomes was generated by CpGAVAS (Liu et al. 2012) and a circular representation was drawn using the online tool OGDRAW (Lohse et al. 2007). The complete chloroplast genome sequence has been submitted to Genbank with the accession number MT663341.

The complete chloroplast genome sequence of *C. chuongtsoensis* is 156,504 bp in length, including two inverted repeat regions (IRa and IRb, each 26,018 bp) separated by a large single copy (LSC) (86,215 bp) region and a small single copy (SSC) (18,253 bp) region. The AT content of the overall chloroplast genome, IR regions, LSC, and SSC are 62.65, 57.01, 64.61, and 69.40%, respectively. The AT content of the two IR regions is lower than those of the SSC and LSC, which is very common in other plants; this phenomenon is mostly attributable to tRNA and rRNA genes (Liu et al. 2019, 2020). The chloroplast genome contains 135 genes in total, including 87 protein-coding genes, 37 tRNAs, 8 rRNAs, and 3 pseudogenes.

The whole genome was used for phylogenetic tree analysis. MAFF v7.427 (Katoh et al. 2005) auto mode was used to align each sequence, and the gaps in the alignment were removed using the program trimAl with ‘-nogaps’ v 1.4 (Capella-Gutierrez et al. 2009). MrBayes v3.2.7 (Fredrik et al. 2012) was used to construct the phylogenetic tree (Figure 1). This study will be useful for further analysis of genetic diversity in *Camellia*.

**Figure 1.**
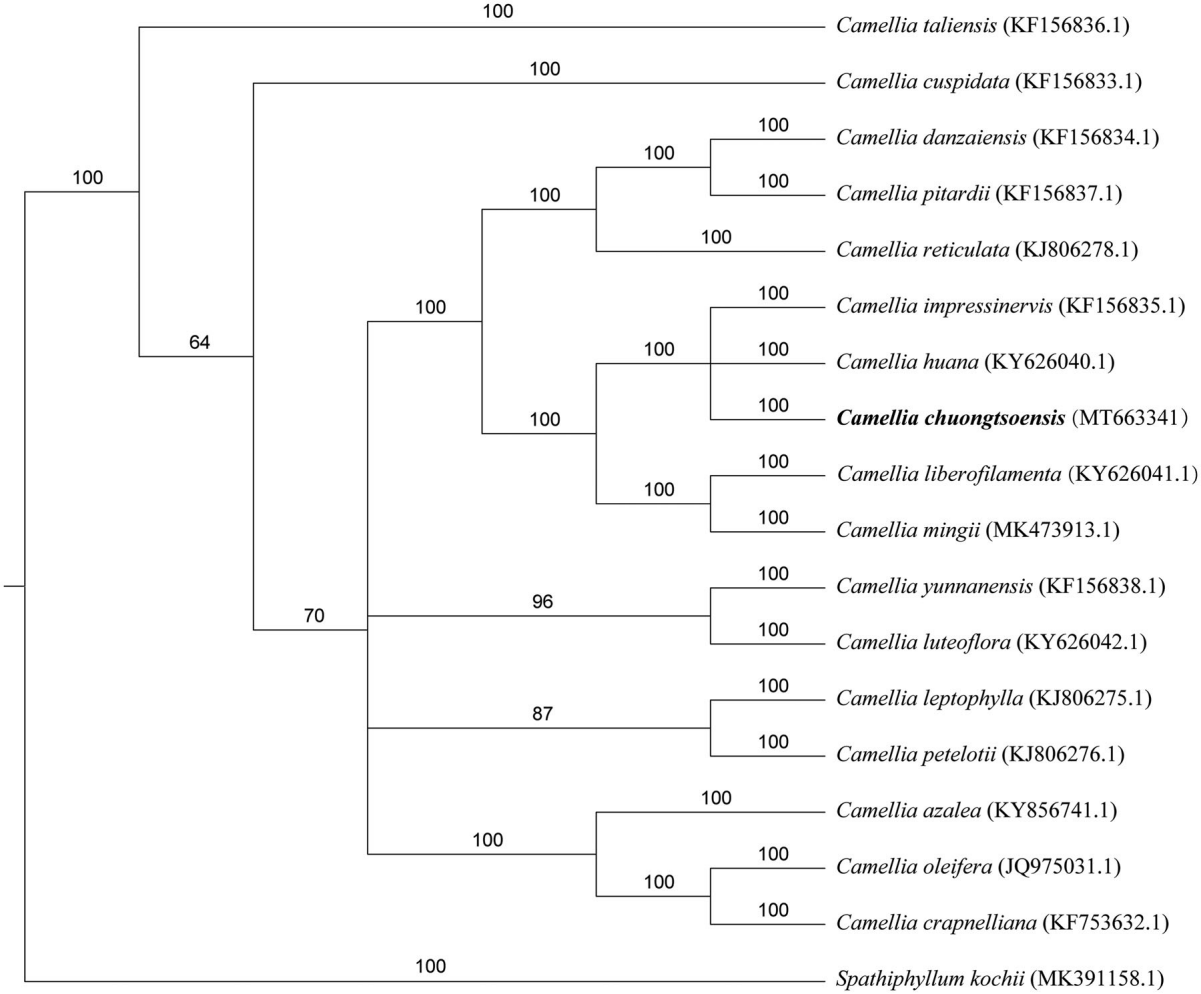
Phylogenetic tree reconstruction of 18 species based on sequences from whole chloroplast genomes. All the sequences were downloaded from NCBI Genbank.

## Author contributions

Performed the experiments investigation, project administration, writing the original draft and data curation: BY.

Prepared the resources: YS, LH, YX, CZ.

Supervised the project and made revisions to the manuscript: XL.

## Data Availability

Data were newly obtained in this study are available in the NCBI under accession number of MT663341 (https://www.ncbi.nlm.nih.gov/nuccore/MT663341).
